# Antimicrobial Resistance in *Pseudomonas aeruginosa* before and during the COVID-19 Pandemic

**DOI:** 10.3390/microorganisms11081918

**Published:** 2023-07-28

**Authors:** Enrica Serretiello, Roberta Manente, Federica Dell’Annunziata, Veronica Folliero, Domenico Iervolino, Vincenzo Casolaro, Alessandro Perrella, Emanuela Santoro, Massimiliano Galdiero, Mario Capunzo, Gianluigi Franci, Giovanni Boccia

**Affiliations:** 1 Clinical Pathology and Microbiology Unit, San Giovanni di Dio and Ruggi D’Aragona University Hospital, 84131 Salerno, Italy; enrica.serretiello@unicampania.it (E.S.); mcapunzo@unisa.it (M.C.); gfranci@unisa.it (G.F.); 2 Department of Experimental Medicine, University of Campania “Luigi Vanvitelli”, 80138 Naples, Italy; manente392@gmail.com (R.M.); federica.dellannunziata@unicampania.it (F.D.); massimiliano.galdiero@unicampania.it (M.G.); 3 Department of Medicine, Surgery and Dentistry “Scuola Medica Salernitana”, University of Salerno, 84084 Salerno, Italy; vfolliero@unisa.it (V.F.); vcasolaro@unisa.it (V.C.); esantoro@unisa.it (E.S.); 4 Department of Public Health and Infectious Diseases, Sapienza University of Rome, 00185 Rome, Italy; iervolino.1886704@studenti.uniroma1.it; 5 Division Emerging Infectious Disease and High Contagiousness, Hospital D Cotugno, 80131 Naples, Italy; alessandro.perrella@ospedaledeicolli.it; 6 UOC Hospital and Epidemiological Hygiene, San Giovanni di Dio and Ruggi D’Aragona University Hospital, 84131 Salerno, Italy

**Keywords:** *Pseudomonas aeruginosa*, nosocomial infections, antibiotic treatment, multidrug resistance, antimicrobial resistance, ESKAPE, COVID-19

## Abstract

*Pseudomonas aeruginosa* (PA) is a major Gram-negative opportunistic pathogen causing several serious acute and chronic infections in the nosocomial and community settings. PA eradication has become increasingly difficult due to its remarkable ability to evade antibiotics. Therefore, epidemiological studies are needed to limit the infection and aim for the correct treatment. The present retrospective study focused on PA presence among samples collected at the San Giovanni di Dio and Ruggi D’Aragona University Hospital in Salerno, Italy; its resistance profile and relative variations over the eight years were analyzed. Bacterial identification and antibiotic susceptibility tests were performed by VITEK^®^ 2. In the 2015–2019 and 2020–2022 timeframes, respectively, 1739 and 1307 isolates of PA were obtained from respiratory samples, wound swabs, urine cultures, cultural swabs, blood, liquor, catheter cultures, vaginal swabs, and others. During 2015–2019, PA strains exhibited low resistance against amikacin (17.2%), gentamicin (25.2%), and cefepime (28.3%); moderate resistance against ceftazidime (34.4%), imipenem (34.6%), and piperacillin/tazobactam (37.7%); and high resistance against ciprofloxacin (42.4%) and levofloxacin (50.6%). Conversely, during the 2020–2022 era, PA showed 11.7, 21.1, 26.9, 32.6, 33.1, 38.7, and 39.8% resistance to amikacin, tobramycin, cefepime, imipenem, ceftazidime, ciprofloxacin, and piperacillin/tazobactam, respectively. An overall resistance-decreasing trend was observed for imipenem and gentamicin during 2015–2019. Instead, a significant increase in resistance was recorded for cefepime, ceftazidime, and imipenem in the second set of years investigated. Monitoring sentinel germs represents a key factor in optimizing empirical therapy to minimize the spread of antimicrobial resistance.

## 1. Introduction

Hospital-acquired infections (HAIs) are a major public health problem with direct and indirect social and economic impacts [1]. According to the Centers for Disease Control and Prevention (CDC), 10 out of 100 patients acquire HAI, with deaths accounting for 87.1% [2,3]. Additionally, HAI contributes to healthcare costs of up to $45 billion [1]. *Pseudomonas aeruginosa* (PA) represents one of the main pathogens responsible for HAIs [4]. Its remarkable ability to colonize a large variety of environments makes it a major player in nosocomial infections [5]. Indeed, this bacterial species contributes to 10–11% of HAI cases, mainly causing respiratory, urinary, and wound infections [6]. The rate of PA infections markedly increased in the ICU, contributing up to 23% of all ward-acquired infections [7]. Healthcare-associated pneumonia (HAP) and ventilator-associated pneumonia (VAP) account for up to 22% of all hospital-acquired infections. Bergin et al. estimated that PA was the cause of 11% of HAP/VAP cases in ICU patients [8]. PA is also the most common cause of nosocomial urinary tract infections associated with catheter use (CAUTI). Indeed, PA is placed at approximately 10% of all CAUTI cases and 16% in the ICU [9]. Furthermore, it is isolated in approximately 8% of cases of chronic wound infections, and its detection is associated with more serious wound outcomes [10]. Numerous intrinsic resistance mechanisms, a high propensity to acquire resistance determinants, and biofilm formation limit therapeutic options, with the development of severe clinical complications in the patient [11,12,13]. Having a large arsenal of antibiotic resistance mechanisms, PA has earned its place on the World Health Organization’s (WHO) list of priority pathogens that pose the greatest threat to human health [14]. A significant impact on the incidence rate of nosocomial infections and the development of multidrug resistance is attributed to the Coronavirus disease 2019 (COVID-19) pandemic [15]. COVID-19 is responsible for severe pneumonia, acute respiratory distress syndrome (ARDS), and higher mortality and morbidity rates [16]. The COVID-19 (pandemic) notably impacted public health, influencing the management of various health issues, including AMR [17]. Several pieces of evidence have underlined a positive correlation between COVID-19 and AMR reasonably due to the large use of empiric antibiotic therapies in patients [18,19,20]. Since the beginning of the pandemic, the improper use of antibiotics, in particular, ceftriaxone and azithromycin, may have favored the spread of resistant microorganisms, compromising the course of hospitalized patients [21]. Despotovic et al. reported a strong increase in bacterial resistance to imipenem, meropenem, and ciprofloxacin in the post-COVID-19 era in Serbia [22]. Moreover, Tiri et al. reported that the acquisition of carbapenem resistance increased from 6.7% in 2019 to 50% in March–April 2020 in Italy. On the other hand, during the COVID-19 pandemic, the improvement of hygiene and contagion-containment measures should have had a positive impact on the transmission of healthcare-associated infections [23]. In light of this information, the present retrospective study investigated the PA presence in the San Giovanni di Dio and Ruggi d’Aragona hospital over an 8-year study period, 2015–2022. In detail, the study focused on recording the frequency and distribution of PA in different biological matrices, including respiratory samples, urine, wound swabs, etc. Furthermore, the antimicrobial resistance variations in the pre-pandemic and pandemic period were investigated. Our findings could lead to the identification of more targeted therapeutic strategies, which may help limit the spread of AMR. Regarding the consciousness of the AMR issue in hospital and care settings, our study will affect the decision of empirical therapy for PA infection. It should be based on knowledge of local epidemiological trends. Our study shows the current situation in our teaching hospital, contributing to the design of new guidelines for the correct use of antibiotics.

## 2. Materials and Methods

### 2.1. Sample Collection and Processing

The database analyzed included data collected from January 2015 to December 2022 at the University Hospital “San Giovanni di Dio e Ruggi d’Aragona” in Salerno, Italy. Samples from patients aged from 0 to 98 years old who were positive for PA growth were included in the analysis. The biological samples to be processed came from different body districts, such as the respiratory tract (bronchial aspirate, sputum, and bronchoalveolar lavage); cutaneous (wound and ulcer swab), urinary (urine), ocular (eye swab), and auricular districts (ear swab); female and male reproductive tracts (vaginal and urethral swabs), nervous (cerebrospinal fluid cultures) and circulatory (blood cultures) systems; and abdominal cavity (peritoneal and ascitic fluid). Furthermore, medical devices were also received (bladder, endotracheal, venous and arterial catheters, ureteral stents, and drainage tubes). The processing of clinical samples was carried out according to the clinical bacteriology guidelines. Blood samples were inoculated in blood culture bottles in volumes of 5–10 mL and 2–3 mL for adult and pediatric patients, respectively, and were incubated in the BACTEC 9240 blood culture automated system (Becton Dickinson Diagnostic Instrument Systems) for up to 5 days (21 days if endocarditis was suspected). In case of positivity, one drop from the positive bottle was stained and observed by microscope and, subsequently, plated on chocolate (incubated in 5% CO_2_ condition), Tryptic Soy Agar, Columbia NaladixicAcid Agar, McConkey, and Sabouraud glucose agar medium (bioMérieux-l’Etoile, Marcy-l’Étoile, France). Respiratory tract samples, wound swabs, and other samples were plated directly on standard bacteriology media. Catheter samples were placed in an enrichment liquid medium and, on the first day, plated only on chocolate. The enrichment liquid medium was seeded on the aforementioned solid media exclusively in case of its turbidity. Urine samples were plated on CPSE chromogen plates (bioMérieux-l’Etoile, Marcy-l’Étoile, France). All plates and liquid media were incubated at 37 °C for 18–36 h. Sample positivity was evaluated and investigated in accordance with the clinical guidelines specific to each typology of the sample [24,25,26].

### 2.2. Isolates Identification and Antimicrobial Susceptibility 

According to the manufacturer’s instructions, bacterial identification and antimicrobial susceptibility tests were performed with Vitek 2 (bioMerieux, Marcy l’Etoile, France). PA suspensions from pure cultures were inoculated in 3 mL of a 0.45% NaCl solution and adjusted to a McFarland standard of 0.5, using a Densicheck (bioMérieux, Marcy l’Etoile, France). The results of antimicrobial susceptibility tests were interpreted according to EUCAST guidelines (https://www.eucast.org/clinical_breakpoints, accessed on 13 February 2022). The antibiotics tested included piperacillin/tazobactam (2/4–48/8 μg/mL), ceftazidime (0.25–32 μg/mL), cefepime (0.25–32 μg/mL), imipenem (1–12 μg/mL), amikacin (2–48 μg/mL), gentamicin (4–32 μg/mL), ciprofloxacin (0.06–1 μg/mL), and levofloxacin (0.12–8 μg/mL) [27].

### 2.3. Data and Statistical Analysis

Microsoft Excel 2019 (Microsoft Corp., Redmond, CA, USA) was used to elaborate the patients’ demographic data (age and sex), the number of strains isolated, and their antimicrobial susceptibility pattern. Chi-squared tests were used to verify the existence of a possible association between the strain’s incidence/the variation in AMR and the period of observation. The Cochran–Armitage trend test was used to verify the existence of a trend in the years. For both tests, a confidence value of alpha equal to 5% was considered. A *p*-value > 0.05 documented the lack of association between variations of pathogen incidence/variation in resistance as a function of time. Conversely, a chi-squared *p*-value < 0.05 documented a significant association, which was further analyzed by the Cochran–Armitage trend test, whereby a *p*-value < 0.05 documented the existence of a significant trend. R software version 4.1.1 was used to perform the statistical analyses [28].

### 2.4. Ethical Consideration Statement

Our retrospective study was based on laboratory data collected from existing databases and was not directly associated with patients. For this reason, approval from the Human Research Ethics Committee was not required for this study.

### 2.5. Limitations

The present study was limited to a single clinical service and is based on a database analysis. While basic patient information, such as demographics and clinical signs were consistently available, more detailed information, including the antimicrobial(s) administered, the time of hospitalization, and the clinical outcomes, was often not documented. A molecular analysis was not performed to determine the genotypes of isolated strains. 

## 3. Results

### 3.1. Prevalence of PA Strains and Distribution by Gender and Biological Sources

In total, 1739 PA strains were isolated from patient samples in 2015–2019 and 1307 in 2020–2022 at the University Hospital “San Giovanni di Dio e Ruggi d’Aragona” in Salerno, Italy. Regarding gender, in both periods, a higher number of PA infections occurred in male patients, revealing a prevalence of 59.1 and 61.5% in the pre-pandemic and pandemic era, respectively (Table 1a,b). In the years 2015–2019, PA was mostly found in the respiratory district, with a prevalence of 33.9%, followed by the urinary tract and skin, with rates of 21.6 and 17%. Cultures, blood and cerebrospinal fluid cultures, medical devices, and vaginal swabs were positive in 12.1, 7.5, 3.7, and 2.6%, respectively. Furthermore, PA was found in 1.6% of other biological materials (Table 2a). In the pandemic period (2020–2022), PA caused 30.4, 19.1, and 18.1% of respiratory, skin, and urinary tract infections, respectively. Furthermore, in 14.6, 10.2, 3.1, 1.3, and 3.3% of cases, culture swabs, hemoculture and cerebrospinal fluid cultures, medical devices, vaginal swabs, and cultures of other biological materials were positive for PA (Table 2b). This variation in the PA distribution in the several matrices could be simply due to a variation in the abundance of the samples.

### 3.2. PA’s Antibiotics-Resistance Profile and Its Trend over Time

**Figure 1:**
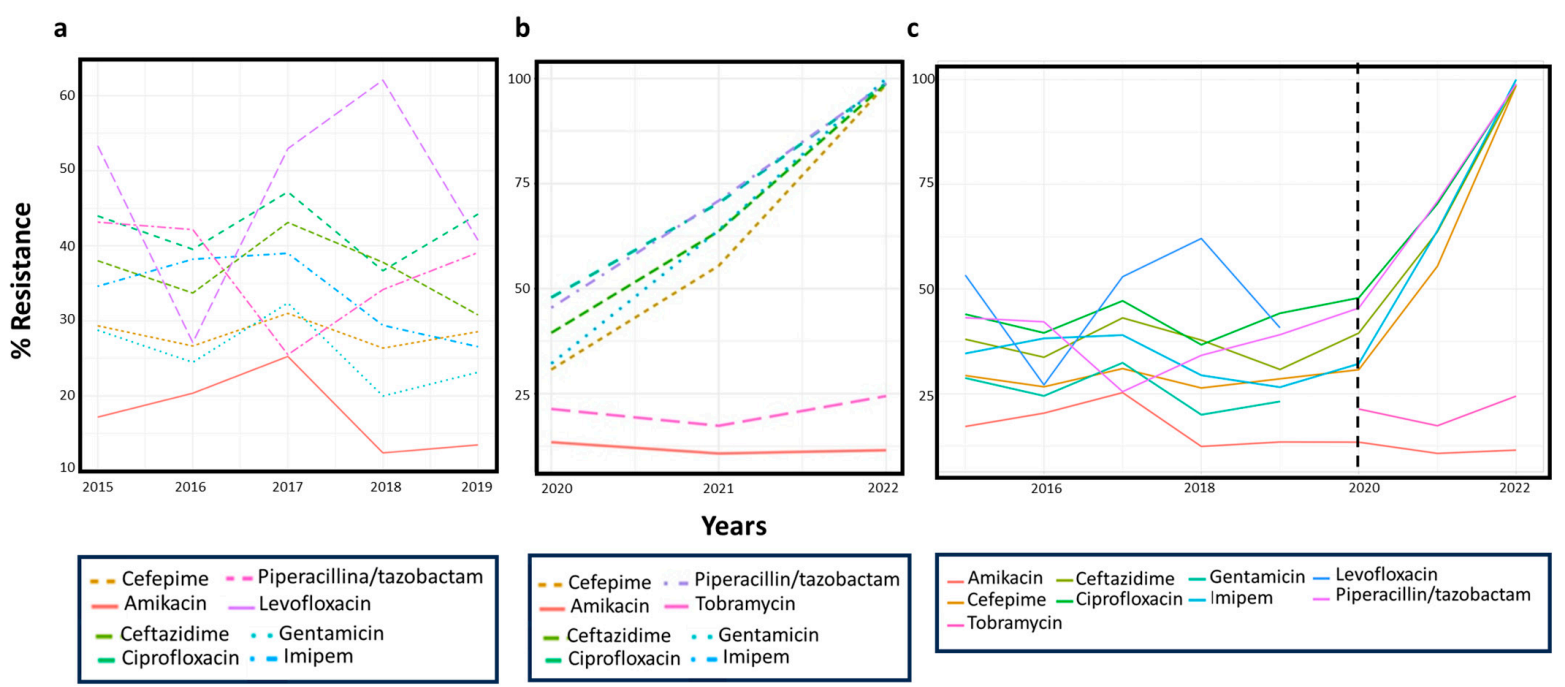
Antibiotics resistance trends in 2015–2019 (**a**), 2020–2022 (**b**), and 2015–2022 (**c**). Dotted black line represents the transition between the period before and during the COVID-19 pandemic.

PA’s susceptibility patterns to the most representative antibiotics and their trends during the eight years investigated are shown in Figure 1a,b. The data reported and discussed below represent the average values in each of the eight years analyzed. During 2015–2019, PA showed the lowest rate of resistance for amikacin (17.2%), followed by gentamicin (25.2%) and cefepime (28.3%). Intermediate resistance rate was recorded for ceftazidime (34.4%), imipenem (34.6%), and piperacillin/tazobactam (37.7%). On the other hand, a resistance rate exceeding 40% was recorded for ciprofloxacin (42.4%) and levofloxacin (50.6%). Resistance to cefepime and ciprofloxacin did not show a relationship between incidence and time (chi-squared *p*-value > 0.05). The existence of a trend over time was also assessed for each antibiotic showing significant variation (Figure 1: *p*-value trend). A significant relationship between incidence and time was found for piperacillin/tazobactam, ceftazidime, and levofloxacin (chi-squared *p*-value < 0.05). Resistance to imipenem and gentamicin showed a significant relationship between incidence and time, with a statistically relevant decreasing trend in 2015–2019 (chi-squared and Armitage trend tests both had a *p*-value < 0.05; Figure 1a and Table 3a). During the pandemic period, PA showed the lowest rate of resistance to amikacin (11.7%), followed by tobramycin (21.1%) and cefepime (26.9%). An intermediate resistance rate was recorded for imipenem (32.6%), ceftazidime (33.1%), ciprofloxacin (38.7%), and piperacillin/tazobactam (39.8%). No resistance rates greater than 40.1% were found for antibiotics tested in the pandemic period. The changes in PA’s resistance to piperacillin/tazobactam, ceftazidime, cefepime, imipenem, tobramycin, and ciprofloxacin over time respected a statistically significant trend (Figure 1b, Table 3b). Antibiotic resistance trends in the 2015–2022 period are shown in Figure 1c.

## 4. Discussion

PA infection management is a very controversial scientific area. Indeed, there is an extensive scientific discussion about the selection of laboratory-tested antibiotics, therapeutic approach relative to the site of infection, and choice of monotherapy or coupled therapeutic regimen [29]. Furthermore, the multiresistance profiles shown by PA often leave little space for the therapeutic approaches currently available [30]. The “Regional system for the surveillance of antimicrobial resistance” (Si.Re.Ar.) program reported 4330 isolates of PA in 2019 from different biological matrices, the most abundant of which were from the respiratory and urinary tracts. This distribution of PA isolates is in line with our data, which show a higher detection rate in the respiratory, urinary, and wound tracts. In agreement with our data, De Rosa et al. identified these districts as the main sites infected with PA at the University Hospital in Heidelberg, Germany [31]. Although the prevalence of PA remained high in the respiratory district in both periods, PA was isolated with different frequencies in wound and urinary tracts. A higher prevalence of PA in infected-wound cases was identified in the pre-COVID-19 period, while PA urinary tract infections were more common in the pandemic period. This could be attributed to the limited admissions to severely ill patients requiring a catheter. Bessa et al. identified PA as one of the main pathogens infecting the respiratory, cutaneous, and urinary tracts [32]. Saeed et al. reported a high frequency of PA in sputum, urine, and wound swab samples [33]. Similarly, Cabot et al. [34] and Sader et al. [35] found a high prevalence of nosocomial respiratory, urinary, and wound infections due to PA. These findings reflected the resistance rates recorded at the University Hospital “San Giovanni di Dio e Ruggi d’Aragona” in Salerno, which showed an amikacin resistance rate (17.2%) lower than for the other antibiotics. The relatively low frequency of PA in blood samples was confirmed in AR-ISS 2020 (“Sorveglianza Nazionale dell’Antibiotico-Resistenza- Istituto Superiore di Sanità”), in which its presence was recorded in 8.2% of samples collected [21,36,37]. Lila et al. showed that, in a tertiary care hospital in Kosovo, during a three-year period, 553 PA isolates were collected, the majority of which resulted from respiratory samples, followed by wounds and blood. Their antimicrobial susceptibility profiles from 2013 to 2015 showed an increase in resistance for cefepime (31.6% to 64.5%), gentamicin (47.2% to 56.6%), amikacin (38.3% to 52.7%), ciprofloxacin (32.8% to 45%), and piperacillin/tazobactam (26.6% to 44.1%), while a decrease in PA’s resistance to ceftazidime (59.8% to 42.0%) and carbapenems (imipenem and meropenem, resistance rates of 29.8% and 18%, respectively) was found [38]. Our data show a decrease in PA’s resistance to imipenem (from 34.6 to 26.6%) and gentamicin (from 28.8% to 23.2%) that was statistically significant over time. The highest resistance rates were found in our study for ciprofloxacin (42.4%) and levofloxacin (50.6%); average rates were found for ceftazidime, imipenem, and piperacillin/tazobactam (from 34.4% to 37.7%); and the lowest rates were found for amikacin (17.2%), gentamicin (25.2%), and cefepime (28.3%). Studies by Bessa, Saeed, Cabot, and Sader et al. showed reduced PA resistance to amikacin, reporting it as the most active antibiotic for the treatment of bacteremia and pneumonia due to PA [32,33,34,35,36]. When grouping the tested antibiotics by class, a statistically significant variation and a decreasing trend were found for all classes, except for aminoglycosides (although *p*-values for this class were very close to significant). The decreasing carbapenems resistance trend aligns with the AR-ISS 2020 reports [37]. The distribution of PA’s resistance to carbapenems was extremely variable in Europe. In 2020, in 4 of the 41 countries reporting data on invasive PA isolates (Denmark, Finland, The Netherlands, and Sweden), rates below 5% were estimated, while 6 countries (Belarus, Bosnia and Herzegovina, Montenegro, the Republic of Moldova, Serbia, and the Ukraine) reported percentages of 50% or more [39]. On the other hand, the data recorded in the present study did not appear to be as alarming as in other areas, where high antimicrobial resistance has been documented. However, the variable distribution of biological matrices examined in each location does not allow for a conclusive comparison. The decreasing trend of imipenem resistance was encouraging and in line with the decrease highlighted by AR-ISS 2020 for 2019, even if an increase in PA’s resistance to carbapenems, by 2% for 2020, was reported in Italy; further studies will be necessary to better understand its long-term trend [40]. During the pandemic period, PA showed a higher susceptibility to amikacin (11.7%), tobramycin (21.1%), and cefepime (26.9%). Resistance rates above 40.1% for the tested antibiotics were not found. A statistically significant increase in resistance over time occurred for piperacillin/tazobactam (resistance % of 39.8), ciprofloxacin (38.7), ceftazidime (33.1), cefepime (26.9), imipenem (32.6), and tobramycin (21.1). No statistically significant resistance trend over time was observed for amikacin. The rates of antibiotic resistance PA in the pandemic period were lower than those recorded in the pre-pandemic period, with values not exceeding 40.1%. Despite this, when analyzing the pandemic period, we recorded an obvious increase in the percentages of resistance during the three years analyzed—alarming data. Several recent studies were published about the effects of the COVID-19 pandemic on AMR spread. Raoofi et al. demonstrated the increase in AMR among Gram-negative bacteria in a retrospective observational study in Southern Iran. Particularly, the average change rate of PA (89%) and *Klebsiella pneumonia* (66.3%) resistance for the reported antibiotics was observed during the COVID-19 pandemic compared to the pre-pandemic [41]. Another retrospective study from April 2019 to April 2021, conducted in an adult ICU at the Hospital for Infectious and Tropical Diseases, Belgrade, Serbia, emerged as COVID-19 changed the landscape of HAIs (HAIs) in intensive care units (ICUs) [22]. In a study conducted in the tertiary hospital of Romania, Coseriu et al. demonstrated alterations in PA susceptibility to carbapenems, pipera-cillin/tazobactam and amikacin before and during COVID-19 (2017-2022), due to appropriate dissemination of antibiotic therapy guides. In particular, they found that the percentage of PA carbapenem- or fluoroquinolone-resistance was lower in 2020–2021 compared with 2018–2019 [42]: these results are in line with the report from the European Centre for Disease Prevention and Control [43]. Several studies are underway to identify new alternative strategies for the treatment of priority bacterial species. These include the evaluation of natural or synthetic antibacterial molecules, chemical modifications of existing molecules, chemical/physical approaches, and antimicrobial synergism, among others [44]. For example, chemically modified norfloxacin salts showed activity against the membrane of *S. aureus* and *P. aeruginosa* [45]. Other alternative approaches include iron chelation, phage therapy, electrochemical scaffolds, inhibition of quorum sensing and bacterial lectins, and, last but not least, vaccine strategy. These could be used alone or in combination with conventional antibiotic treatments [46]. A popular approach relies on the synergism of antibiotics, such as the imipenem–relebactam combination or combinations of fosfomycin with other molecules with which it synergizes in vitro (e.g., aztreonam, cefepime, or ceftazidime) [47]. It has been shown that mutations conferring resistance to fosfomycin occurred at a lower rate when fosfomycin was administered in combination with ceftazidime–avibactam [48]. An inappropriate or delayed therapy could affect both the patient’s outcome and the length of hospitalization. Since choosing an appropriate empirical treatment is rather problematic, the management of critically ill patients with PA-MDR infections must be implemented and supported by accurate and rapid sensitivity tests and the definition of resistance mechanisms. Therefore, a laboratory equipped for rapid identification of the pathogen and the characterization of its resistance profile using antibiogram panels, including the latest generation molecules; the choice of suitable and timely therapeutic approaches, the monitoring of resistance trends, and the implementation of new alternative strategies are key points for the correct management of patients presenting PA infections [49,50]. Several studies show conflicting evidence on the correlation between hospital infections caused by MDR pathogens and COVID-19. This happens because a wide use of empirical antibiotic therapy in patients affected by COVID-19 could be related to an increase in resistance in microorganisms [51,52]. However, other authors have found a reduction in infections caused by MDR organisms in this period when compared to the pre-pandemic period. They related it to the containment and prevention measures adopted in response to COVID-19. In detail, Ipek et al. noted a reduction in the incidence of *K. pneumoniae* and no cases of PA or *E. faecium* in their pediatric intensive care unit. This result could be correlated to an increase in the rate of hand-hygiene practices during the pandemic period [53]. Similarly, Reynolds et al. reported a significant reduction in the prevalence of MDR bacterial infections in the pandemic period when compared to the pre-pandemic rate [54]. Despite the different scientific relevance, the conclusion of our study did not indicate any apparent difference in the PA incidence rate and susceptibility profile during the pre- and post-pandemic era. Further studies will be needed to investigate the PA trend in the other hospitals of the region in order to implement regional epidemiological comparative studies.

## 5. Conclusions

AMR is a serious global public health problem, which is estimated to cause more than 10,000 deaths in the world by 2050, more than the deaths caused by cancer and HIV/AIDS [55]. The intrinsic properties of PA, along with its resistance-acquired strategies, make it insensitive to several antibiotics. Therefore, the development of novel antibacterial strategies is needed. Indeed, PA is listed among the “critical priority” pathogens on the “WHO priority pathogens list for R&D of new antibiotics” [56]. As PA is responsible for 10–15% of nosocomial infections worldwide and has a major impact on healthcare costs, its correct therapeutic management is of fundamental importance [54]. Hospitals should have clear local infection control guidelines in place. In fact, current efforts must be aimed at monitoring not only its frequency and distribution over different samples but also its resistance profile and related fluctuations over time. In this way, suitable empirical therapies could prevent PA infections. In addition, medical and non-medical personnel should receive periodic training in infection control procedures. Due to conflicting evidence deriving from pre- and mid-pandemic COVID-19 period studies, further research is needed to investigate possible correlations between pathogens responsible for major hospital infections, especially respiratory infections, and COVID-19. 

## Figures and Tables

**Table 1:** Gender distribution per year in the years examined: (**a**) 2015–2019 and (**b**) 2020–2022 N., number of patients; %, percentage of female or male patients relative to total patients.

**(a)**						
**Years**	**2015**	**2016**	**2017**	**2018**	**2019**	**Tot.**
Sex	**N. (%)**	**N. (%)**	**N. (%)**	**N. (%)**	**N. (%)**	**N. (%)**
F	104 (35.6%)	143 (41.7%)	143 (49.5%)	147 (40.1%)	175 (39.1%)	712 (40.9%)
M	188 (64.4%)	200 (58.3%)	146 (50.5%)	220 (59.9%)	273 (60.9%)	1027 (59.1%)
Tot. for Year	292 (100%)	343 (100%)	289 (100%)	367 (100%)	448 (100%)	1739 (100%)
**(b)**						
**Years**	**2020**		**2021**	**2022**		**Tot.**
Sex	**N. (%)**		**N. (%)**	**N. (%)**		**N. (%)**
F	112 (31.55%)		170 (35.8%)	221 (44.3%)		503 (38.5%)
M	243 (68.5%)		305 (64.2%)	256 (53.7%)		804 (61.5%)
Tot. for Year	355 (100%)		475 (100%)	477 (100%)		1307 (100%)

F, female; M, males; Tot., total.

**Table 2:** Distribution of clinical isolates in 2015–2019 (**a**) and 2020–2022 (**b**) by material/site of infection collected. Culture swabs were derived from other body districts.

**(a)**												
**Years**	**2015–2019**		**2015**		**2016**		**2017**		**2018**		**2019**	
**Samples**	**Isolates n.**	**Isolates %**	**Isolates n.**	**Isolates %**	**Isolates n.**	**Isolates %**	**Isolates n.**	**Isolates %**	**Isolates n.**	**Isolates %**	**Isolates n.**	**Isolates %**
Respiratory	590	33.9%	114	39.0%	98	28.6%	112	38.8%	127	34.6%	139	23.2%
Wound swabs	375	21.6%	58	19.9%	73	21.3%	60	20.8%	80	21.3%	104	31.0%
Urinary	296	17.0%	33	10.3%	60	17.5%	45	15.6%	78	21.8%	80	17.9%
Cultural swab	210	12.1%	30	11.3%	46	13.4%	33	11.4%	37	10.1%	64	14.3%
Blood and Liquor culture	130	7.5%	27	9.2%	31	9.0%	13	4.5%	25	6.8%	34	7.6%
Catheters	65	3.7%	16	5.5%	16	4.7%	13	4.5%	10	2.7%	10	3.1%
Vaginal swabs	45	2.6%	4	3.4%	12	3.5%	10	3.5%	5	1.4%	14	2.2%
Others	28	1.6%	10	1.4%	7	2.0%	3	1.0%	5	1.4%	3	0.7%
**Tot. Isolates**	1739	100%	292	100%	343	100%	289	100%	367	100%	448	100%
**(b)**												
**Years**	**2020–2022**		**2020**		**2021**		**2022**					
**Samples**	Isolates n.	Isolates %	Isolates n.	Isolates %	Isolates n.	Isolates %	Isolates n.	Isolates %				
Respiratory	397	30.4%	107	30.1%	166	34.9%	124	26.0%				
Urinary	249	19.1%	66	18.6%	86	18.1%	97	20.3%				
Wound swabs	237	18.1%	62	17.5%	70	14.7%	105	22.0%				
Cultural swab	191	14.6%	56	15.8%	70	14.7%	65	13.6%				
Blood and Liquor culture	133	10.2%	36	10.1%	50	10.5%	47	9.9%				
Others	43	3.3%	12	3.4%	22	4.6%	9	1.9%				
Catheters	40	3.1%	12	3.4%	9	1.9%	19	4.0%				
Vaginal swabs	17	1.3%	4	1.1%	2	0.4%	11	2.3%				
**Tot. Isolates**	1307	100%	355	100%	475	100%	477	100%				

Tot., total.

**Table 3:** Antibiotics resistance trends in 2015–2019 (**a**) and 2020–2022 (**b**). Statistically significant values are indicated in bold.

(**a**)														
**Antibiotics**	**2015**		**2016**		**2017**		**2018**		**2019**		**Total Years**		***p*-Value**	***p*-Value Trend**
	**R**	**N. Test**	**R**	**N. Test**	**R**	**N. Test**	**R**	**N. Test**	**R**	**N. Test**	**R**	**N. Test**		
Pip./taz.	43.2%	285	42.2%	332	25.5%	196	34.2%	357	39.1%	445	37.7%	1615	**0.001**	0.11
Ceftazidime	38.0%	292	33.7%	344	43.1%	283	29.9%	365	30.8%	448	34.4%	1732	**0.01**	0.126
Cefepime	29.4%	293	26.7%	345	31.0%	287	26.4%	307	28.6%	147	28.3%	1379	0.69	0.794
Imipenem	34.6%	286	39.0%	341	39.0%	282	29.4%	306	26.6%	143	34.6%	1358	0.02	0.0221
Amikacin	17.2%	285	20.4%	319	25.3%	273	12.4%	354	13.5%	415	17.2%	1646	<0.0001	0.089
Gentamicin	28.8%	292	24.5%	343	32.4%	284	20.0%	365	23.2%	449	25.2%	1733	0.00	0.0326
Ciprofloxacin	44.0%	291	39.5%	344	47.2%	284	37.8%	365	44.2%	450	42.4%	1734	0.09	0.931
Levofloxacin	53.3%	15	53.3%	30	52.9%	34	62.1%	29	40.7%	54	50.6%	162	0.01	0.312
(**b**)														
**Antibiotics**	**2020**			**2021**			**2022**			**Total Years**			***p*-Value**	***p*-Value Trend**
	**I**	**R**	**N. Test**	**I**	**R**	**N. Test**	**I**	**R**	**N. Test**	**I**	**R**	**N. Test**		
Pip./taz.	11.7%	40.1%	349	43.4%	40.2%	468	60.2%	39.2%	475	41.0%	39.8%	1292	0.0188	0.017
Ceftazidime	13.2%	34.3%	356	53.0%	30.0%	474	66.4%	35.3%	467	46.9%	33.1%	1297	0.0497	0.0143
Cefepime	9.7%	27.8%	299	55.5%	24.7%	474	70.3%	28.5%	478	50.2%	26.9%	1251	0.00249	<0.001
Imipenem	5.7%	30.3%	300	48.6%	32.8%	475	66.3%	33.7%	478	45.1%	32.6%	1253	<0.001	<0.001
Amikacin	1.5%	13.3%	332	0.4%	10.7%	466	0.0%	11.6%	476	0.5%	11.7%	1274	0.553	0.278
Tobramycin	0%	21.4%	248	0.0%	17.4%	380	0.0%	24.4%	401	0.0%	21.1%	1029	<0.001	<0.001
Ciprofloxacin	13.2%	41.6%	356	48.7%	36.1%	474	60.5%	39.1%	478	43.3%	38.7%	1308	0.124	0.0422

Pip./taz., piperacillin/tazobactam.

## Data Availability

The data are contained within the article.
